# A Hybrid Framework for Intrusion Detection in Healthcare Systems Using Deep Learning

**DOI:** 10.3389/fpubh.2021.824898

**Published:** 2022-01-12

**Authors:** M. Akshay Kumaar, Duraimurugan Samiayya, P. M. Durai Raj Vincent, Kathiravan Srinivasan, Chuan-Yu Chang, Harish Ganesh

**Affiliations:** ^1^BrainSightAI, Bangalore, India; ^2^Department of Information Technology, St. Joseph's College of Engineering, Chennai, India; ^3^School of Information Technology and Engineering, Vellore Institute of Technology, Vellore, India; ^4^School of Computer Science and Engineering, Vellore Institute of Technology, Vellore, India; ^5^Department of Computer Science and Information Engineering, National Yunlin University of Science and Technology, Douliu, Taiwan; ^6^Service Systems Technology Center, Industrial Technology Research Institute, Hsinchu, Taiwan

**Keywords:** healthcare, network security, neural networks, artificial intelligence, big data, deep learning

## Abstract

The unbounded increase in network traffic and user data has made it difficult for network intrusion detection systems to be abreast and perform well. Intrusion Systems are crucial in e-healthcare since the patients' medical records should be kept highly secure, confidential, and accurate. Any change in the actual patient data can lead to errors in the diagnosis and treatment. Most of the existing artificial intelligence-based systems are trained on outdated intrusion detection repositories, which can produce more false positives and require retraining the algorithm from scratch to support new attacks. These processes also make it challenging to secure patient records in medical systems as the intrusion detection mechanisms can become frequently obsolete. This paper proposes a hybrid framework using Deep Learning named “ImmuneNet” to recognize the latest intrusion attacks and defend healthcare data. The proposed framework uses multiple feature engineering processes, oversampling methods to improve class balance, and hyper-parameter optimization techniques to achieve high accuracy and performance. The architecture contains <1 million parameters, making it lightweight, fast, and IoT-friendly, suitable for deploying the IDS on medical devices and healthcare systems. The performance of ImmuneNet was benchmarked against several other machine learning algorithms on the Canadian Institute for Cybersecurity's Intrusion Detection System 2017, 2018, and Bell DNS 2021 datasets which contain extensive real-time and latest cyber attack data. Out of all the experiments, ImmuneNet performed the best on the CIC Bell DNS 2021 dataset with about 99.19% accuracy, 99.22% precision, 99.19% recall, and 99.2% ROC-AUC scores, which are comparatively better and up-to-date than other existing approaches in classifying between requests that are normal, intrusion, and other cyber attacks.

## Introduction

Network intrusion recognition is challenging since the attacks evolve daily because of new technologies, frameworks, and software. In 2020, the number of cyber-attacks increased by 17%, in that 77% were targeted attacks, with attackers' main targets being personal data and credentials. Attacks on organizations aimed mainly at stealing private user data. These metrics show a vital backdrop in modern-day cyber-attack detection and prevention. Moreover, the healthcare industry is increasing, and most hospitals are integrating e-healthcare systems to meet the patients' needs as soon as possible. Hospitals must maintain the Electronic Health Records (EHR) and Patient Records or Personal Health Records (1) since these details contain a patient's medical data required to infer a diagnosis and treatment. The vast development in the Internet of Things (IoT) has led to a boom in smart medical devices and systems. These edge devices can contain patient records, which must be kept secure and accurate at all times. Any change or corruption in these details can lead to wrong diagnosis and treatment, causing the fatality of the patient. Therefore, to ensure cyber-safety in healthcare systems, there is a need for up-to-date and advanced Hybrid Intrusion Detection Systems.

The common goal of any Intrusion Detection System is to recognize, flag, and log/block intrusion attacks by identifying any malicious network activity (1, 2). Most of the existing real-time software for IDS uses a rule-based approach like signature-based detection, stateful protocol analysis, and statistical packet analysis. Primarily the IDS classifies a request into benign and malicious, benign being regular requests, and malicious being anomalous or intrusion requests. The IDS are also specifically designed to identify a specific set of attacks like DDoS.

However, Artificial Intelligence-based systems come with specific backlogs; the rate of false positives can be overwhelmingly frequent compared to actual threats. The algorithms may also be biased toward particular attacks based on class imbalance and features present in the dataset used for training the algorithm. Inaccurate and biased intrusion detection systems don't stand a chance in protecting patient records as they may falsely flag an attack as benign or a benign request as malicious. The issue with the existing intrusion detection systems is that they are trained on outdated or old datasets like KDD-Cup'99, making the system vulnerable to the latest attacks and leading to leakage and illegal modification of EHRs. Traditional Machine Learning algorithms such as K-Nearest Neighbors (3) and Logistic Regression usually need repeated fine-tuning and updating parameters to adapt the algorithm to new types of attacks that may require frequent retraining, which is computationally expensive and time-consuming. Ensemble learning (4) and deep learning-based (5) approaches have proven better because of their adaptability and ease of fine-tuning or pruning on new data. Faster and efficient algorithmic techniques are still vital for such critical applications as time complexity is a significant overhead in medical and IoT-based systems. A state-of-the-art, present-day attack-oriented, faster, and efficient methodology is required for Intrusion Detection Systems to safeguard patient records in e-healthcare.

**Figure 1:**
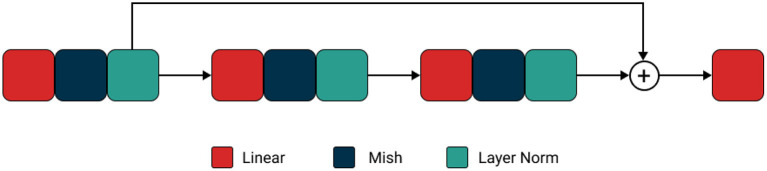
Neural network of ImmuneNet architecture.

This paper proposes a new hybrid framework for intrusion detection using deep learning for healthcare systems named “ImmuneNet.” We have benchmarked its performance against various machine learning algorithms on the Canadian Institute for Cybersecurity's IDS 2017 (6), IDS 2018 (7), Bell DNS 2021 (8) datasets. These are realistic datasets containing more than 17 types of the latest cyberattacks, which would be beneficial in implementing real-time deep learning-based intrusion detection systems and achieving high accuracy. A data-centric approach was used to promote class balance (9) and extensive feature selection processes like recursive feature elimination (10, 11), which can help better perform models with fewer parameters. Over five types of machine learning algorithms were compared: Logistic Regression, Decision Trees, Random Forests, Extreme Gradient Boosting Trees (12), and a custom neural network architecture named “ImmuneNet” as seen in Figure 1. ImmuneNet obtained the highest accuracy of almost 99.19% and other vital metrics from all the experiments. The neural network architecture contains around 830,000 parameters, making the fine-tuning process faster and deployment-friendly for medical devices and systems.

Our experiments were based on several data-centric and algorithmic approaches to create a lightweight, fast, high-performance classifier without compromising accuracy. We have divided our paper into the following sections based on different schemes:

1. The current state-of-the-art methodologies and existing research for implementing intrusion detection systems in healthcare and general.
2. Comparison between KDD Cup99, CIC-IDS variants, and CIC Bell DNS 2021 datasets to know in detail about the attack types, size, number of samples, and state of relevance.
3. Extensive feature selection, oversampling, and hyper-parameter optimization techniques involved in training, validating, and testing the algorithms on the three datasets.
4. Comparison of multiple machine learning algorithms, namely logistic regression, ensemble methods such as decision trees, extreme gradient boosting trees, and a custom neural network architecture named “ImmuneNet” using various metrics like accuracy, loss, precision, recall, F1, and loss.
5. A detailed discussion on the proposed system's performance, advantages, and limitations.

## Related Works

Our literature survey primarily focused on analyzing the data preprocessing and algorithmic approaches to derive new novel solutions for developing a sound intrusion detection system. There are tons of cyber vulnerabilities to cope within the medical sector as these attacks threaten patients' healthcare and put them in jeopardy. Tervoot et al. (13) performed a scoping review by categorizing and analyzing these attacks and found 18 solutions, each fitting at least one of the categories of intrusion detection and prevention, communication tunneling, or hardware protections. Thamilarasu et al. (14) proposed an intrusion detection system for the internet of medical things based on Wireless body area networks (WBAN). They simulated a hospital network topology and performed detailed experiments for various subsets of the Internet of Medical Things, including wireless body area networks and other connected medical devices. The system performs best at 99.6 and 98.2% accuracy for network and device-level intrusion detection, respectively.

Subas et al. (4) proposed an IDS with multiple Machine Learning techniques with some complex attack data. They concluded that the Bagging ensemble algorithm and Random Forest perform better with 97.67% accuracy than other classifier models. Hence, a Smart E-Healthcare System could be built on top of this system to ensure better cyber security. The Internet of Medical Things (IMoT) has its drawbacks as technical advancements progress since any security and privacy issues exist. Therefore, Priya et al. (5) developed a deep neural network (DNN) for constructing an effective IDS to classify and predict unforeseen cyberattacks. They also carried out practical feature engineering in hyperparameter selection. The proposed DNN model performs better than the existing machine learning approaches, with an increase in accuracy by 15% with an overall accuracy of 99.7 % on NSL-KDD. Šabić et al. (15) proposed an anomaly detection system that focuses on the system's ability to detect anomalies in heart rate data. They used five algorithms to fit the data, two of them were unsupervised, and the rest were supervised to detect the anomalies. They concluded that random Forest and ensemble methods had above 99% accuracy to model such systems effectively.

Hady et al. (16) proposed and built a real-time Enhanced Healthcare Monitoring System (EHMS) that monitors the patients' biometrics and collects network flow metrics. Then this system applies different machine learning methods for training and testing the dataset against these attacks. Hence by collecting their data dynamically, they developed a robust intrusion detection system. by analyzing in 10-fold accuracy score comparison, they noted that the SVM algorithm performed better than the rest with an accuracy of about 92.44%. Nguyen et al. (17) used data fusion as an integral part of multidisciplinary research to design an intrusion detection system. Their proposed model involves a decision-based fusion model with different processes such as initialization, preprocessing, Feature Selection, and multimodal classification to detect intrusions effectively. The proposed model offers maximum accuracy of 99.21%, precision of 98.93%, and a detection rate of 99.59%. Iwendi et al. (18) proposed an intrusion detection system in the Security of Things (SoT) paradigm for smart healthcare and will continue to impact medical infrastructures. Their study used the NSL-KDD dataset on RF, Naive Bayes (NB), and logistic regression classifiers for machine learning. To optimize the functionality of their approach, they used a weighted genetic algorithm, and Random Forest was coupled to generate achieved a high detection rate and neglect false positives and true negatives. The combination of their genetic algorithm and RF models achieved an accuracy rate of 98.81%.

Yeng et al. (3) conducted a systematic review of various machine learning algorithms and data resources to assess them across multiple criteria. They have also evaluated and analyzed the design considerations of the methods toward mitigating false positives. Their comprehensive study determines the associated challenges in using the algorithms and how they can be overcome. Their study suggests that most of the existing literature for intrusion systems in healthcare uses K-Nearest Neighbors. Mahdavifar et al. (8) have proposed a method to classify benign, phishing, spam, and malware-based domains using DNS traffic analysis with the K-Nearest Neighbors algorithm. They have used the CIC Bell DNS 2021 dataset, which is balanced to 60/50 and 97/3%, achieving an F1 score of around 94.8 and 99.4%, respectively. Seth et al. (19) built a unique framework based on creating an ensemble by ranking the detection ability of different base classifiers to identify various types of attacks. They have experimented with numerous algorithms and achieved 96.65% accuracy with neural networks on the CIC-IDS 2018 dataset and have also given a deep analysis of the metrics on classwise performance. Benedetto Serinellia et al. (20) analyses open-source intrusion detection datasets by validating them to detect well-known zero-day attacks. They have built their predictors based on KDD99, NSL-KDD, and CIC-IDS-2018 datasets, giving a comparative case study among them in their research work. They achieved over 99.97% accuracy on the NSL-KDD datasets. Thilagam et al. (21), on the other hand, proposed an Intrusion detection system on a more complex basis using RCNN (Recurrent Convolutional Neural Networks) using the culmination of CNN and LSTM (Long Short Term Memory) on the KDD Cup 99 and CIC-IDS 2018 datasets. They achieved over 94% accuracy on both datasets with the same model.

Class Imbalance is another critical attribute to be considered for building a novel and efficient architecture. Zuech et al. (22) analyzed web attacks using random undersampling ratios under various ensemble learning algorithms and discussed class balance's significant importance. They observed that undersampling at different ratios could have a drastic effect on the model's performance and achieved an accuracy of about 94.01% accuracy on CIC IDS 2018 dataset using RCNN. Yu et al. (23) had proposed a packet byte-based CNN, called PBCNN, which focuses on the statistical features of network traffic, giving another insight on the importance of network traffic and its hierarchical nature. PBCNN obtained 99.99 % accuracy on the CIC IDS 2017 and CIC IDS 2018 datasets. Gopalan et al. (9) carried out a balancing approach and surveyed the CIC-IDS-2018 dataset. They also researched the impact of bias and class imbalances in the CIC-IDS-2018 dataset. As observed, signature-based intrusion detection approaches prove to be the weaker counterpart of various methods.

Most of the research on Intrusion detection mechanisms was based on the ensemble learning approach. Fitni et al. (24) made comparisons with seven single classifiers to identify the most appropriate basic classifiers for ensemble learning; they compared the accuracy metrics of tested architectures and made a cumulative study on it. They also used effective feature selection techniques like filter methods and Spearman rank's correlation and used only 23 features to achieve 98.8% accuracy on the CIC IDS 2018 dataset. We used the MISH activation function in our custom neural network architecture. Misra (25), the author of MISH. Discussed in detail its scope and found that. Mish outperformed other activation functions like Leaky ReLU on YOLOv4 with a CSP-DarkNet-53 backbone on average precision. Hua et al. (26) proposed using the LightGBM ensemble method, which proved to be quite a popular and efficient ensemble method for Intrusion Detection. By achieving 98.37% accuracy, the study also compared their approach with other ensemble methods and traditional machine learning algorithms to prove its efficiency.

Catillo et al. (27) presented an autoencoder-based bi-level anomaly detector, which used a specific Artificial Neural Network to reconstruct their vectorized input on the CIC IDS 2017 and 2018 datasets with a maximum detection rate of 99.2 %. It seems to be an overwhelming algorithmic approach to a relatively simple problem with minimal feature space. Khan and Kim (28) have proposed a Convolutional auto-encoder (Conv-AE) based Intrusion Detection System using a heterogeneous dataset. The Conv-AE algorithm proves to efficiently combine the advantages of traditional state-of-art approaches and identifies malicious attacks in the case of both anomaly and misused-based ID systems. They have also mentioned the distributed and big-data-friendly processing pipelines for training their algorithms and achieved 98.2 % accuracy. Meryem and Ouahidi (29) overcame the limitations of anomaly and misuse detection by using a hybrid approach. They have employed the K-Nearest Neighbors on the KDD-Cup99 dataset. They had achieved more than 98.77% and an Area Under the Curve (AUC) score of 0.58, which is sub-optimal. Thakkar and Lohiya (30) briefly discuss CIC-IDS-2017 and CIC-IDS-2018 datasets by analyzing their features and iterating them across many Machine Learning and Deep Learning models. They had suggested that the CIC IDS datasets were significantly better than NSL KDD because of the type of attacks, data capturing techniques, and attack infrastructure.

According to Gao et al. (31), an adaptive and ensemble system to the same anomaly-based approach proved better. Using the KDD Cup99 dataset, they used multiple algorithms such as KNN, DNN, random forest, and to architect an ensemble adaptive voting algorithm. They compared the algorithms and found that the Adaboost model achieved 99.99% accuracy. Reviewing and understanding the dataset is vital for building innovative and novel solutions. Chawla et al. (32) in their paper, had attempted to build an efficient intrusion detection system that was anomaly-based, using Recurrent Neural Networks like Gated Recurrent Units rather than the vanilla LSTM networks. However, convolutions and recurrent methods can be computationally intensive and time-consuming compared to other methodologies applied for non-sequential tabular data. They have obtained an accuracy of only around 81% on the ADFA Intrusion Detection dataset. Anomaly-based detection has its disadvantages, supporting the opinion of Feras et al. (33) proposed a hybrid model that leverages the functionalities of SVMs and decision trees. Using the CIC IDS 2018 and KDD Cup 99 datasets, they have accuracy of 97.881 and 99.982%, respectively. Wankhede and Kshirsagar (34)–“DoS Attack Detection Using Machine Learning and Neural Network” uses the dataset CIC IDS 2017 dataset and uses Random Forest and Multi-Layer Perceptron approach. The Random Forest algorithm provides more optimized results than Multi-Layer Perceptron from their experiments, obtained around 99% accuracy on the CIC-IDS 2017 datasets.

Mubashar et al. (1) created a framework for an IoT-based medical data archival system by combining a novel block chain-based technique for medical data encryption with an optimization algorithm. Loshchilov and Hutter (35) proposed Stochastic Gradient Descent with Warm Restarts, from which we have used the Cosine annealing learning rate scheduling technique as an optimization strategy in our experiments. Glorot and Bengio (36) had proposed a new weights initialization technique for neural networks that leads to better convergence and performance of the model. Sharma and Yadav (10) in their paper have proposed recursive feature elimination technique for feature selection and have achieved an accuracy of about 99% accuracy for DOS attacks and other attacks using Decision Trees on KDD Cup 99 dataset. Correlation based feature selection was proposed by Thaseen et al. (37) in their paper as a feature selection method, and obtained an overall accuracy of 97.9 % with an artificial neural network on the NSL-KDD dataset (38–42).

From our observation, most of the research used the KDD-Cup99, and the approaches done with CIC-IDS datasets support the latest intrusion attacks. They are performing better while also supporting better classes of attacks. The features in the CIC-IDS 2017 and 2018 datasets are quite the same. They can also be used together with multiple feature selection techniques and sampling techniques to produce better results for the classifiers. The Intrusion Detection mechanism must be trained on the latest cyber-attack repositories to be up-to-date hence protecting the patient records without any compromise. It is also essential that the algorithm is not biased toward benign or malicious classes, which may cause high false positive rates (43–50). Hence, a balanced dataset should be used for training the IDS mechanism. Our paper has proposed the methodologies followed for feature selection, class balancing, data preparation, and preprocessing under multiple sub-sections in section Methodology. The results, comparison for the algorithms, and discussion on the pros and cons of the proposed system are discussed under sections Results and Discussion, respectively.

## Methodology

Our approach deals with building a hybrid intrusion detection mechanism that can support the latest cyberattacks without following any traditional rule-based methods. The IDS should protect electronic Health Records (EHR) from any type of attack. An algorithm that can be pruned or fine-tuned to adapt to new attacks is crucial since recent attacks are evolving daily. We experimented with a new dataset by the Canadian Institute for Cybersecurity that supports over 15 types of latest intrusion attacks and compared the same with the widely available datasets for intrusion detection. A statistical feature selection process has been done on the dataset to extract the most valuable features that can boost the performance of the classifiers. We also dealt with the class imbalance of the dataset to not make our model biased toward particular positives or negatives. We compared five machine learning algorithms: Logistic Regression, Decision Trees, random forests, XGB (12), and Artificial Neural Network. We have created a new Artificial Neural Network architecture that surpassed performance compared to the other algorithms obtaining ~99.2 % accuracy. We conducted our experiments on a Linux machine running Ubuntu 21.04 OS with 32 GB RAM and 8 GB NVidia GTX 1080 GPU to use distributed training of our algorithms to obtain results faster. We used Python and popular data science libraries like NumPy, Pandas, Scikit-Learn, and TensorFlow to implement the data processing and algorithms. We have discussed all the aspects of our methodology in detail in the following subsections.

### Dataset Comparison

Datasets used for Intrusion Detection and Coping purposes evolve a lot with time since there is a perennial and dynamic change in cybersecurity threats and new attacks rise around the horizon day in and out. The most traditional dataset used for this purpose is the KDD Cup 99 dataset, which is proven to have a lot of anomalies. NSL-KDD is the dataset suggested to solve some of the problems of the KDD 99 dataset. It removes all the redundant records in the KDD 99 train set, ensuring no duplicate records in the proposed test sets. NSL-KDD's KDD training set contains 22 attack types, and KDDTest data contains additional 17 attack types. It has 41 attributes and one class attribute, respectively. Another dataset commonly explored for this use case is The ISCX dataset 2012 by the Information Security Center of Excellence at the University of New Brunswick. It contains over 2 million traffic packets categorized under 20 features. It covers attacks such as Brute Force SSH, HTTP DoS, DDoS and Infiltrating attacks. CIC analyzed these attacks under regular traffic rates on seven successive days, and they logged the corresponding metrics. It is one of the most up-to-date datasets.

CIC IDS 2017–2018 is the dataset we prefer for our Intrusion Detection System as it contains benign and the most up-to-date common attacks, which resembles the actual real-world data (PCAPs). It also includes the results of the network traffic analysis using CICFlowMeter with labeled flows based on the timestamp, source and destination IPs, source and destination ports, protocols, and attack. It is a labeled dataset containing a total number of 84 features, including their corresponding traffic status. Moreover, CIC IDS 2017 covers various attacks such as Brute Force Attack, HeartBleed Attack, Botnet, DoS Attack, Distributed DoS (DDoS) Attack, Web Attack, and Infiltration Attack. The total number of records in the dataset is 3,830,743. The benign traffic lists around 2,273,097 records, whereas the malicious samples are 5,57,646 in number. The CIC IDS 2018 dataset consists of the same attack classes but with a large number of samples for benign and malicious classes. It consists of around 13,484,708 benign samples and 2,748,235 malicious samples.

Apart from this, we have also used the latest CIC Bell DNS 2021 dataset, which contains up-to-date real-time DNS-related data that can be useful for flagging a particular request as benign, spam, phishing, and malware. CIC created this dataset in collaboration with Bell Canada and Cyber Threat Intelligence, Canada. There were 32 features in the dataset, formed from DNS-statistical features and lexical features. The classes in the dataset were further grouped into 400,000 benign entries and 13,011 malicious entries, from which they balanced the classes by grouping 20,000 benign scans and 13,011 malicious scans together. Table 1 displays the meta-data such as dataset size, number of entries, provider, and attack classes in detail for KDD Cup 99, CIC IDS 2017–2018, and CIC Bell DNS 2021.

**Table 1:** Comparison of KDDCup99, CIC IDS variants, and CIC Bell DNS 2021 datasets.

	**KDD Cup 99**	**CIC IDS 2017**	**CIC IDS 2018**	**CIC Bell DNS 2021**
Year	1999	2017	2018	2021
Provider	University of California, Irvine	Canadian Institute for Cybersecurity, University of New Brunswick	Canadian Institute for Cybersecurity, University of New Brunswick	Canadian Institute for Cybersecurity, University of New Brunswick + Bell, Canada + Cyber Threat Intelligence, Canada
Size (GB)	0.75	1.06	5.81	0.287
Number of samples	494,020	2,830,743	16,232,943	413,011
Classes	DoS, U2R, R2L, Probe + sub-attacks	DDoS, DoS slowIoris, BotNet, Port Scan, SQL Injection + 7	DDoS, DoS slowIoris, BotNet, Port Scan, SQL Injection + 10	Benign, Spam, Phishing, Malware

### Feature Selection

There were 82 features present in both CIC IDS 2017 and 2018 datasets. Table 2 displays some of the features present in the dataset. Out of these, there were a lot of similar features that were highly related to each other, like the forward packet's total length and header length of packets. Deriving features with less correlation would help the algorithm learn diversely as they comparatively have lesser variance. Since both CIC IDS 2017 and 2018 had the same features, we calculated the correlation by combining both datasets as they belong to the same distribution.

**Table 2:** Features in CIC IDS 2018 with corresponding correlation values and *p*-value based significance.

**S. No**	**Feature name**	**Correlation value**	***p*-value**
1	active_mean	0.9086	>0.05
2	Protocol	0.7950	<0.05
3	subflow_fwd_pkts	0.9992	>0.05
4	flow_pkts_s	0.2177	<0.05
5	bwd_pkts_s	0.2349	<0.05
6	pkt_size_avg	0.9410	>0.05

**Figure 2:**
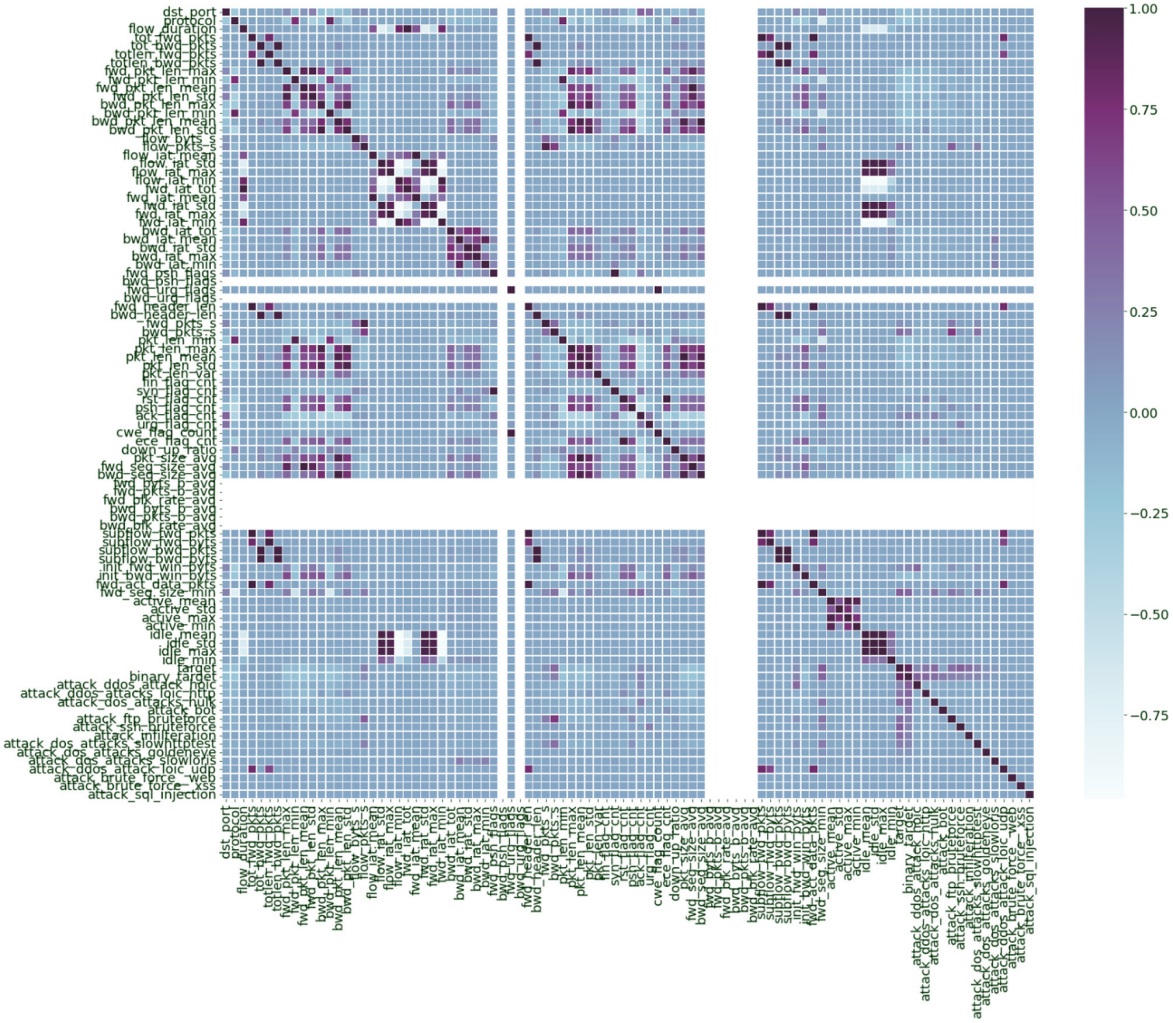
Correlation plot of the features in the combined CIC IDS 2017 and CIC IDS 2018 dataset.

We have employed Correlation-based Feature Selection (CFS) (37). By plotting the correlation matrix of the features (Figure 2), we analyzed which features were highly correlated to elucidate which ones were independent. Features with high correlation are more linearly dependent, and they tend to have the same effect on the dependent variable. So, when we observe two features with a relatively high correlation, we can drop one of those features, which reduces the computation complexity and dataset size to be processed. Around 56 features were derived that were correlated <75%. Further, we used recursive feature elimination (11, 37) by using a logistic regression classifier and selected 38 features based on their significance with an alpha value (*p*-value) of 0.05. Table 2 represents some of the features in the dataset with corresponding correlation scores and *p*-value based significance.

Table 3 displays some of the selected features from the CIC Bell DNS 2021 dataset. The dataset was subject to multiple feature engineering techniques in processing the raw PCAP files into DNS statistical, lexical, and third-party features, as Mahdavifar et al. (8) suggested. We have used all 32 derived features from the dataset and have applied different preprocessing techniques discussed in section Data Preparation.

**Table 3:** Example features in CIC Bell DNS 2021 dataset.

**S. No**	**Country**	**ASN**	**Entropy**	**Name_Server _Count**	**Domain_Age**
1	NL	20,415.0	3.1547	0.0	1,735
2	PL	198,414.0	2.6250	4.0	5,117
3	US	46,606.0	3.0079	4.0	510
4	AU	45,638.0	3.4058	17.0	307
5	FR	12,876.0	2.2893	25.0	603

### Improving Class Balance

**Figure 3:**
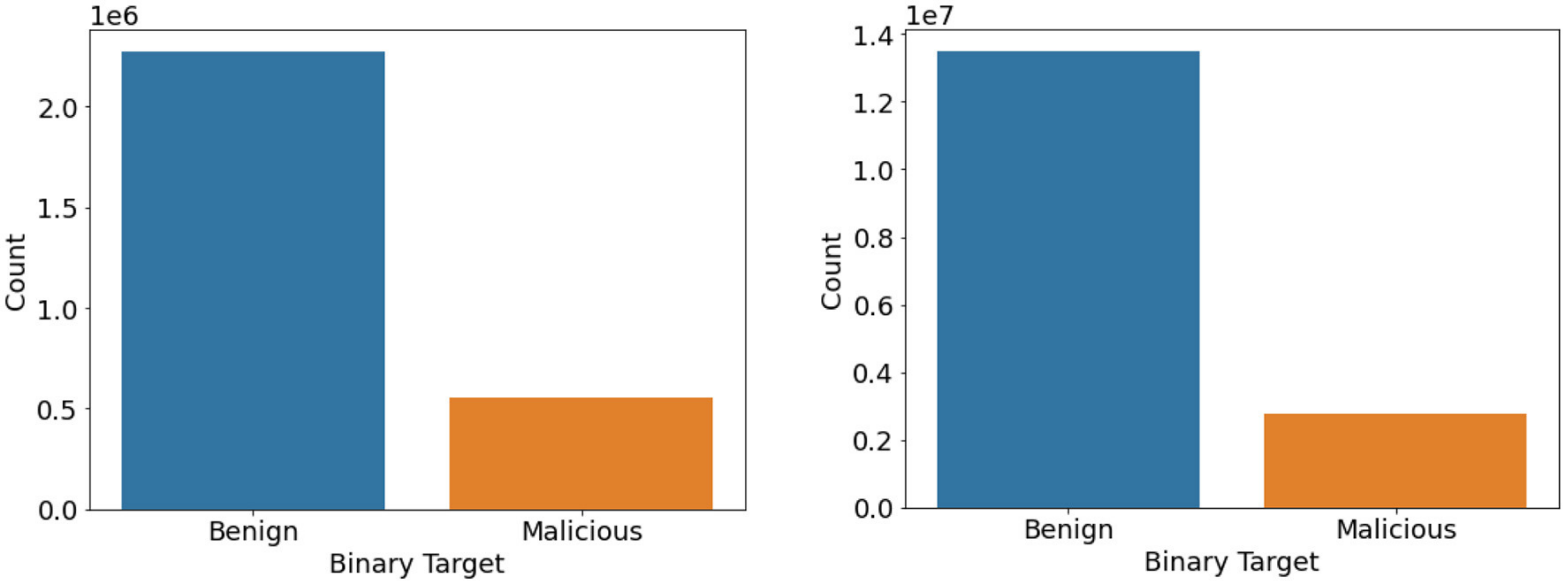
Binary class count plot of CIC IDS 2017 (left) and 2018 (right) datasets.

**Figure 4:**
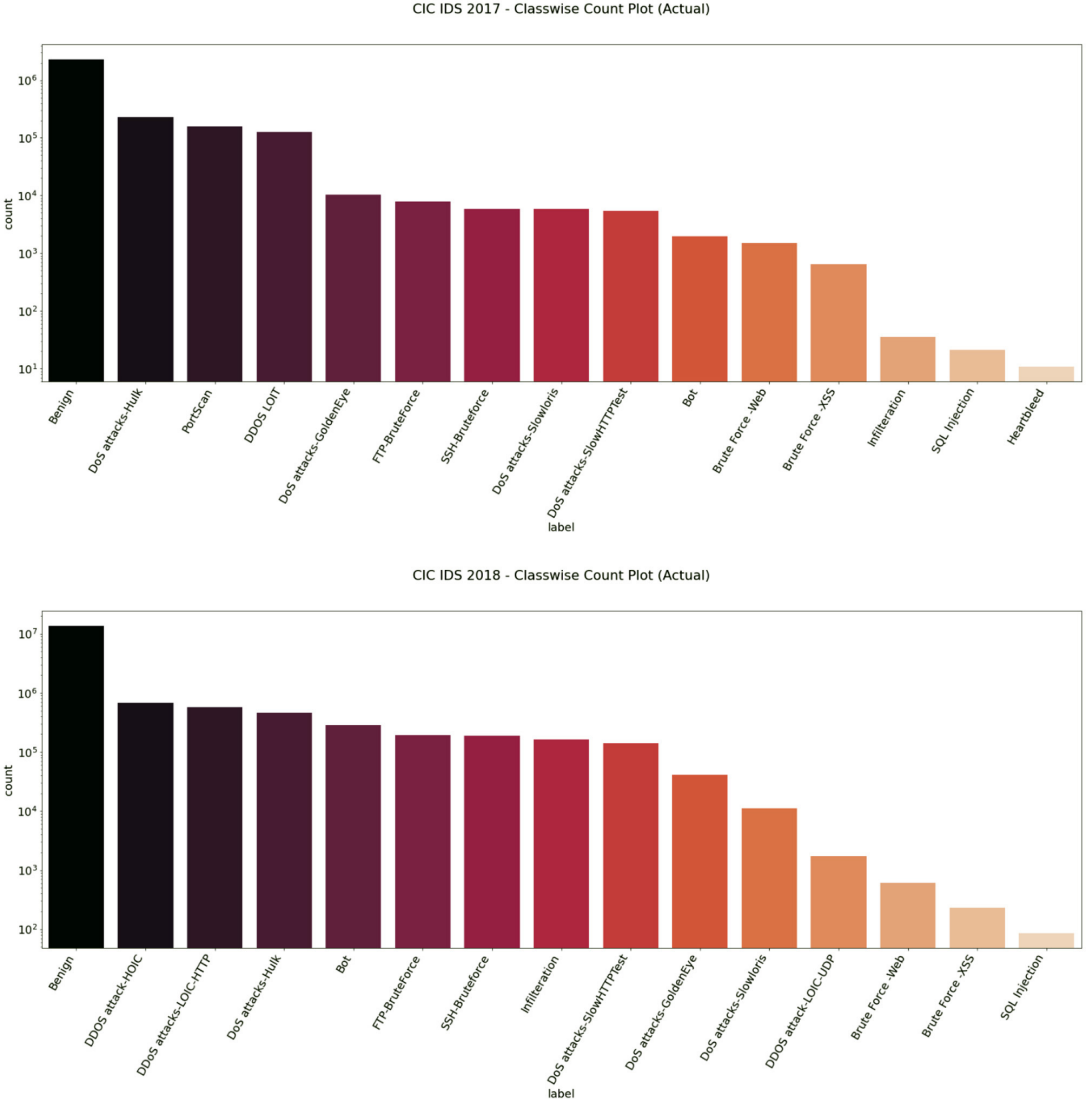
Classwise count plot of CIC IDS 2017 (top) and 2018 (bottom) datasets.

Balanced datasets lead to good accuracy and performance of the classifiers. We found that the dataset had a heavy class imbalance by plotting the count of benign and intrusion samples. The number of benign samples was higher than the number of intrusion samples. One way to overcome this quickly would be to under-sample it. Since we have samples <100 for a few classes like SQL Injection in the CIC IDS 2018 dataset as referred to in Figures 3, 4, under-sampling the imbalance dataset would still yield an imbalanced dataset with fewer samples. It may cause a bias toward higher-sample labels while training any algorithm.

**Figure 5:**
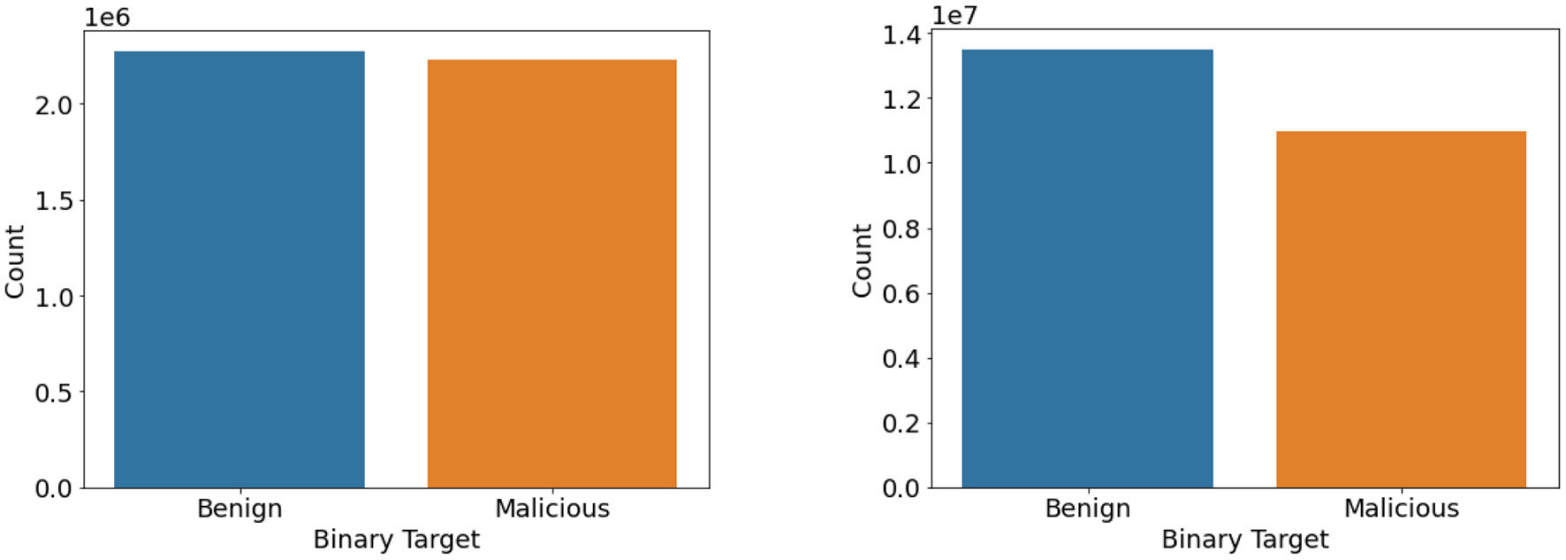
Binary class count plot of oversampled CIC IDS 2017 (left) and 2018 (right) datasets.

**Figure 6:**
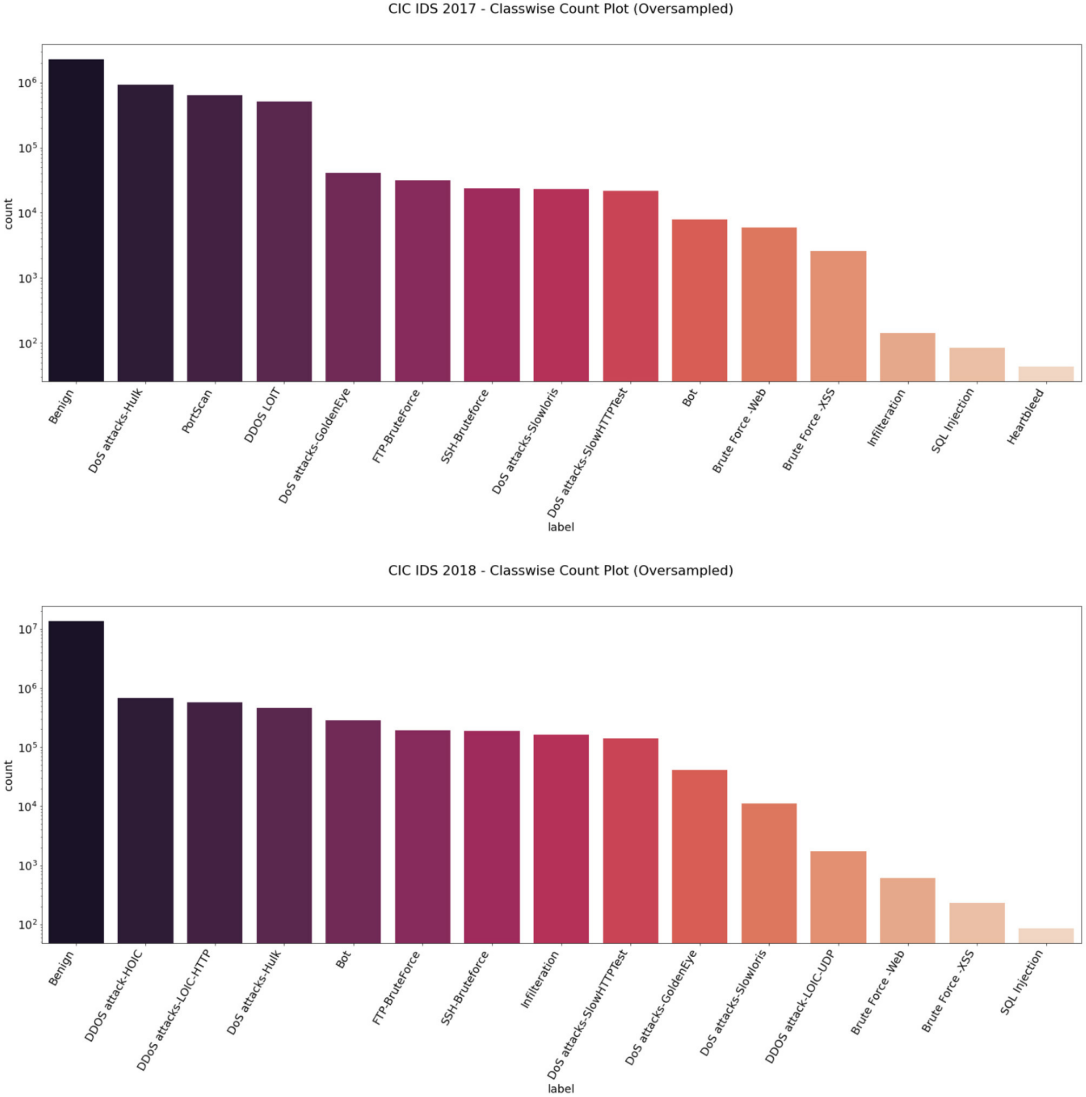
Classwise count plot of oversampled CIC IDS 2017 (top) and 2018 (bottom) datasets.

We then used the Synthetic Minority Oversampling Technique algorithm to oversample the CIC IDS 2017 and 2018 datasets by a factor of 3 only for the intrusion class. Imbalance classification involves developing models on datasets that have a severe class imbalance. Synthetic Minority Oversampling Technique or SMOTE algorithm is a data augmentation technique that selects features in close feature space. It creates as many synthetic examples for minority classes, making them more effective in learning the decision boundary and performing better. SMOTE uses K-Nearest Neighbors to select points near the neighbor of each data point randomly and generates synthetic instances to upsample the minority class. SMOTE oversampling is effective because the new synthetic samples generated from the minority class have relatively close feature space to existing samples. In this way, we were able to get an almost balanced dataset, leading to better performance of the model. Figures 5, 6 represent the oversampled binary count plot and classwise count plot of the CIC IDS 2017 and 2018 datasets.

**Figure 7:**
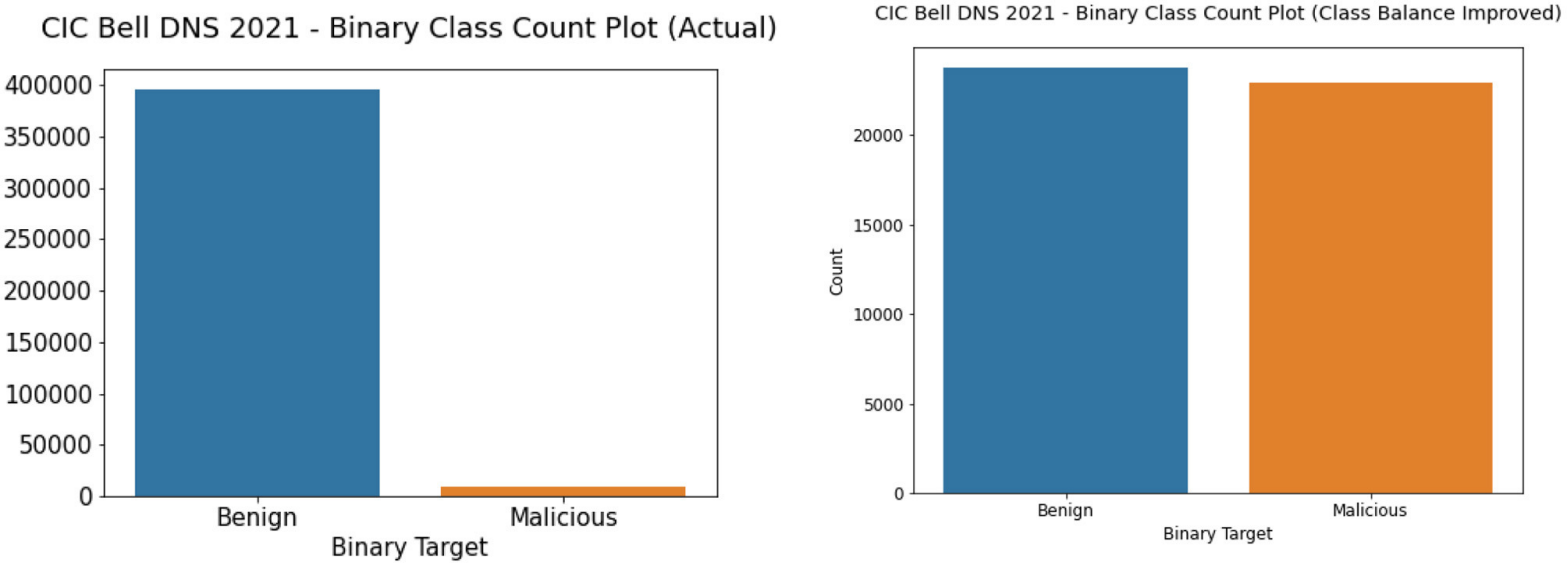
Binary class count plot of actual (left) and class balance improved (right) CIC Bell DNS 2021 dataset.

**Figure 8:**
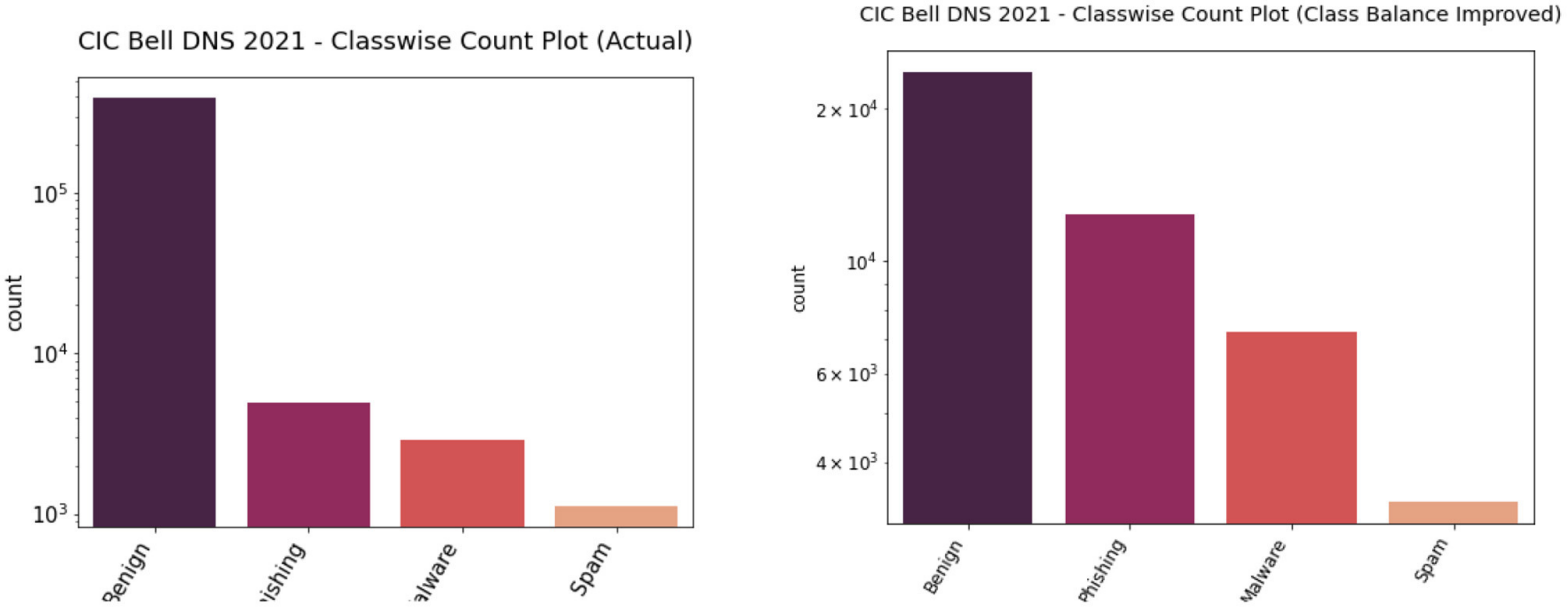
Classwise count plot of actual (left) and class balance improved (right) CIC Bell DNS 2021 dataset.

We performed both undersampling and oversampling for the CIC Bell DNS 2021 to promote class balance. The dataset had a heavy class imbalance with 400,000 benign samples and only 13,011 malicious samples. Random shuffling and selection were performed on the dataset to gather only around 25,000 benign samples. Apart from this, SMOTE oversampling was done on the rest of the three classes: spam, phishing, malware. We also removed entries that contained null values since they were not usable. After this process, we had a balanced dataset with 23,716 benign samples and 22,929 malicious samples. Figures 7, 8 illustrate the binary and classwise count plots of samples in the CIC Bell DNS 2021 dataset before and after the class balance improvement process.

Table 4 shows the number of samples and benign to malicious sample ratio before and after class balance improvement for CIC IDS 2017, CIC IDS 2018, and CIC Bell DNS 2021 datasets.

**Table 4:** Data sample numbers and percentage before and after oversampling CIC IDS 2017, 2018, and CIC Bell DNS 2021 datasets.

	**Actual**		**Balanced**		**Actual Percentage**		**Balanced Percentage**	
	**Benign**	**Malicious**	**Benign**	**Malicious**	**Benign**	**Malicious**	**Benign**	**Malicious**
CIC IDS 2017	2,273,097	557,646	2,273,097	2,230,584	80.3	19.7	50.47	49.53
CIC IDS 2018	13,484,708	2,748,235	13,484,708	10,992,940	83.07	16.93	55.09	44.91
CIC Bell DNS 2021	400,000	13,011	23,716	22,929	96.84	3.16	50.84	49.16

### Data Preparation

The dataset was made publicly available by the Canadian Institute for Cybersecurity (CIC). The CIC Bell DNS 2021 dataset's processed CSV files were downloaded from their website. We performed standard data cleaning to remove null values and encode categorical text such as domain names, countries, and states with numbers. The CIC IDS 2017 and 2018 datasets were downloaded from their AWS S3 bucket using the AWS CLI and split into multiple CSV files based on the type of attack. We then combined these CSV files as a single data frame during runtime to save memory for further preprocessing as the entire dataset was more than 7 GB. The dataset was subject to multiple feature selection and sampling processes as mentioned in the previous sections to promote the most valuable features for classification to achieve better accuracy. We combined the attack columns into three feature columns: labels as the attack's name, binary targets denoting whether the packet is benign or malicious, and target column representing the id of which attack had occurred. Later this data was z-scored using the standardization formula (1). Standardization of the data leads to faster and more accurate convergence of the machine learning models.


$$\begin{array}{l} y\;=\;\frac{\left(x-\bar{x}\right)}{\sigma } \end{array}$$

We followed a stratified cross-validation split with a 30% test size on all three datasets while running our algorithmic experiments. The cross-validation split yielded a 70% training set, 15% validation set, and 15% testing set for every fold with a random shuffling of the data entries. Since the split was stratified, we contained the same benign to malicious samples ratio in all three sets for each dataset.

### Machine Learning Approaches

We experimented with five machine learning algorithms inspired by different pieces of contemporary machine intelligence literature whose methods proved to work well for tabular data and critical applications. Logistic Regression is a statistical learning algorithm used to model the probability of an event happening or not. The logistic function or sigmoid function approximates the output values from a multinomial linear equation to predict whether a particular class is occurring. We used L2 regularization and “lbfgs” solver to optimize the algorithm. Equation 2 defines the logit calculation by summation over the dot product of inputs and weights added to bias, which is then applied inside sigmoid, equation 3, to get a probability-like value between 0 and 1, which denotes the occurrence of a class.


$$\begin{array}{l} \;z=\;w^{T}x+b\\ \sigma \left(z\right)=\frac{1}{1+e^{-\left(z\right)}} \end{array}$$

$$\begin{array}{l} \text{Binary Cross entropy Loss}\\ \;=-\frac{1}{\begin{array}{c} output\;\\ size \end{array}}\overset{\begin{array}{c} output\\ size \end{array}}{\underset{i=1}{\sum }}y_{i}\;.\;\log\hat{y_{i}}+\left(1-y_{i}\right).\;\log\left(1\;-\hat{y_{i}}\right) \end{array}$$

Binary Cross Entropy or BCE (4) is used as the cost function with L2 regularization for the optimization process using gradient descent.

Further, we used Decision trees with the ID3 implementation and Gini criterion. We also ran Random forests with 100 trees and criteria as Gini, equation 5. This algorithm establishes the outcome based on the predictions of the decision trees, which uses the information gain, equation 6, as an optimization metric.


$$\begin{array}{r} Gini:\;I_{G}\left(f\right)=\overset{m}{\underset{i=1}{\sum }}f_{i}\;\left(1-f_{i}\right)\\ Information\;gain:\;I_{E}\left(f\right)=\overset{m}{\underset{i=1}{\sum }}f_{i}\;{\log_{2}\;f}_{i} \end{array}$$

Extreme Gradient Boosted Trees (XGB) (12) have recently gained momentum for their outstanding results on different kinds of tabular data. With regularization techniques and effective pruning of the trees, only significant splits with positive information gain are retained. We observed that the above methods, namely Extreme Gradient Boosted Trees, Random Forests, and Decision Trees, worked better with given use cases than Logistic Regression because ensemble models use multiple independent learners from which the entire predictions are derived.

**Figure 9:**
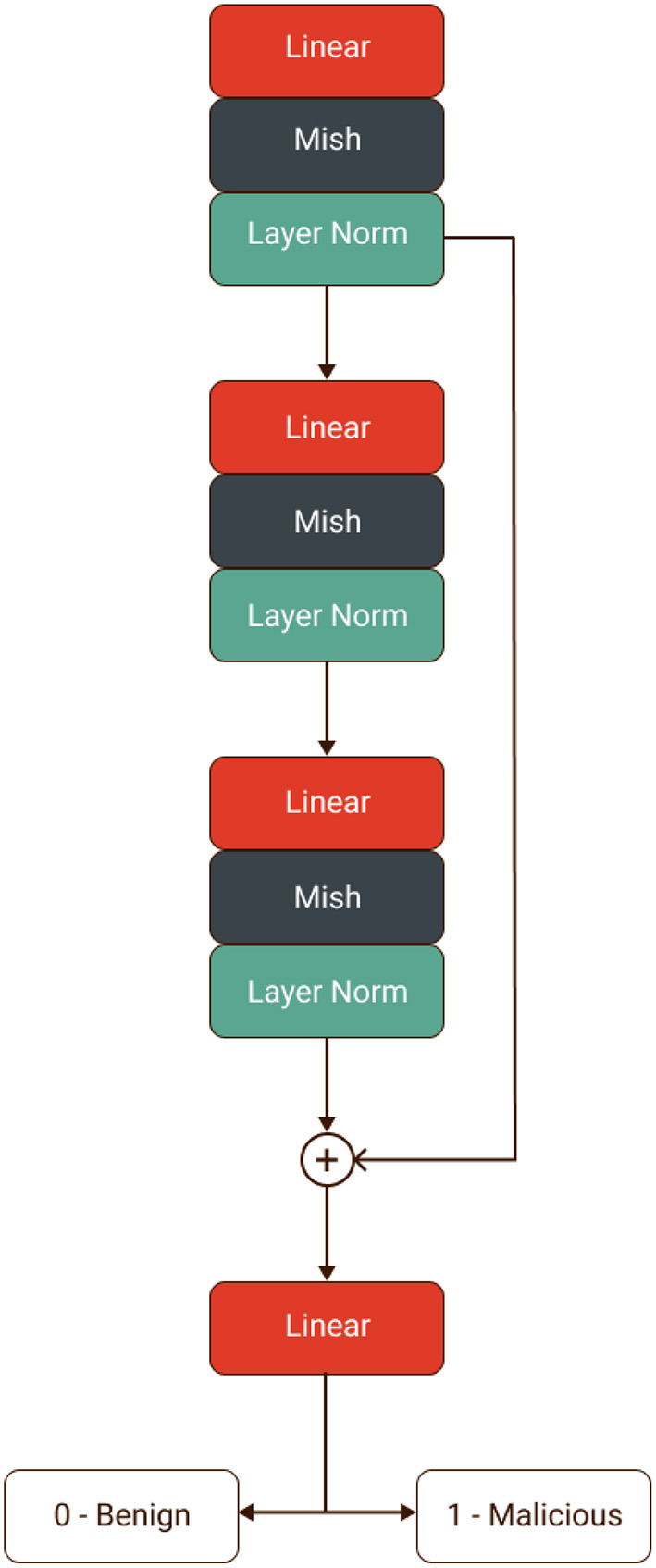
ImmuneNet architecture.

Finally, we built our own Artificial Neural Network named the “ImmuneNet” that uses simple linear projections followed by mish activation and layer normalization with a residual operation between the first and the final block. Figure 9 displays the neural network architecture of ImmuneNet.

The first and last linear layer has 512 neurons, and the hidden layer has 784 neurons. Since there were fewer features for all three datasets, we used a minimal number of neurons in the layers. The number of neurons per layer was set arbitrarily and increased by a factor of 2 after observing the performance of the earliest models by using a grid search with different hyper-parameter settings for the hidden layer units. The first and the last layer are connected with a residual (38) operation, and hence they have the same number of neurons. Equation (7) defines the perceptron or linear equation, which is chained with the mish (25) activation (8) followed by the layer normalization (39) (10), creating a forward block for the immune net. We have used Sharma and Yadav (10) technique for the neural network's weights initialization for training all three datasets. Table 5 describes the hyperparameters we used for ImmuneNet and its optimization for all three datasets with corresponding feature dimensions. The number of samples used for training, validating, and testing the algorithms is mentioned below in Table 6.

**Table 5:** Hyperparameter details for ImmuneNet for CIC IDS 2017, CIC IDS 2018, and CIC Bell DNS 2021 datasets.

**Hyperparameters**	**Values as per dataset**		
	**CIC IDS 2017**	**CIC IDS 2018**	**CIC bell DNS 2021**
Input feature dimension	38	38	32
Batch size	64	64	64
Adam optimizer	β_1_ = 0.9, β_2_ = 0.99, ε = 1e-7	β_1_ = 0.9, β_2_ = 0.99, ε = 1e-7	β_1_ = 0.9, β_2_ = 0.99, ε = 1e-7
Learning rate	1e-4	1e-4	3e-4
Additional optimization strategies	Cosine annealing learning rate scheduling, early stopping	Cosine annealing learning rate scheduling, early stopping	Cosine annealing learning rate scheduling, early stopping
# Epochs	15	15	15
# Units in input FC layer	512	512	512
Input FC layer activation	Mish	Mish	Mish
# Units in hidden FC layer 1	784	784	784
FC layer 1 activation	Mish	Mish	Mish
# Units in hidden FC layer 2	512	512	512
FC layer 2 activation	Mish	Mish	Mish
# Units in output FC layer (binary classification)	1	1	1
Output FC layer activation	Sigmoid	Sigmoid	Sigmoid

**Table 6:** Number of samples in training, validation, and testing set for CIC IDS 2017–2018, and CIC Bell DNS 2021 datasets.

	**Number of samples in the training set**	**Number of samples in the validation set**	**Number of samples in the test set**	**Total**
CIC IDS 2017	3,152,567	675,552	675,552	4,503,681
CIC IDS 2018	17,134,354	3,671,647	3,671,647	24,477,648
CIC Bell DNS 2021	32,652	6,996	6,997	46,645

We used the Adam optimization (40) set to a learning rate of 1e-4 with cosine annealing (36) technique for learning rate scheduling and binary cross-entropy (4) as the cost function. The hyper-parameters for the optimizer except for the learning rate, like beta1, and beta2 have been set with values as suggested by the authors of Adam optimizer (40). The learning rate will be changed after every few iterations based on the validation loss obtained, with the help of the cosine annealing (36) learning rate scheduler. The cosine function is used to vary the lower rate after each epoch. The objective of cosine annealing is to prevent the model from saturating after reaching a local minima and varying the learning rate using the cosine function to periodic lower learning rates to converge at an optimal point like the global minima. The model was trained with a batch size of 64 for 15 epochs with an early stopping mechanism which stops the training process when the model's performance starts to saturate, which acts as a regularization technique. We have avoided larger batch sizes because models with larger batch sizes and higher learning rates can cause unstable training, bad convergence, and lead to different asymptomatic test metrics. The results of all the algorithms mentioned above are discussed in detail under section Results.

Linear Layer:


$$\begin{array}{l} y\;=\;w^{T}.x+b \end{array}$$

Mish Activation:


$$\begin{array}{l} f\left(x\right)\;=\;x\tanh\left(\ln\left(1+e^{x}\right)\right) \end{array}$$

Layer Normalization


$$\begin{array}{r} {\hat{x}}_{i,l}\;=\;\frac{x_{i,k}-\mu_{i}}{\sqrt{\sigma_{i}^{2}}+\in }\\ y_{i}+\gamma {\hat{x}}_{i}+\beta \equiv \;{LN}_{\gamma ,\beta }\left(x_{i}\right) \end{array}$$

## Results

### Evaluation Metrics

The following metrics for evaluating our machine learning models can help derive more significant insights into our various algorithms' performance and bias:

Confusion Matrix (Table 7) is a matrix that summarizes the performance of the given algorithm on the classification. Since the dataset is not entirely balanced, using measures like accuracy alone can be misleading.

**Table 7:** Confusion matrix for binary classification.

	**Class 0**	**Class 1**
Class 0	TP	FP
Class 1	FN	TN

Accuracy, equation (11), or correct rate is how right the model is for the given classification problem.


$$\begin{array}{l} Accuracy\;=\;\frac{TP+TN}{TP+TN+FP+FN} \end{array}$$

Precision (12) is the ratio of true positives to all the positives, i.e., the sum of true positives and false positives.


$$\begin{array}{l} Precision\;=\;\frac{TP}{TP+FP} \end{array}$$

Recall (13) is the ratio between true positives and the sum of true positives and false negatives.


$$\begin{array}{l} Recall\;=\;\frac{TP}{TP+FN} \end{array}$$

The F1 (14) score provides the balance between the precision and recall, suggesting how similar the predicted set is to the true set.


$$\begin{array}{l} F1\;=\;\frac{2^{*}\text{Precision}*\text{Recall}}{Precision+Recall}\;=\;\frac{2^{*}\text{TP}}{2^{*}\text{TP}+FP+FN} \end{array}$$

ROC Curve is the rate of true positives against false negatives describing how well a binary classifier can classify between two classes. The area of the region under the ROC curve defines the performance of the binary classifier. The model performs better when it has a high AUC score (15) and performs the lowest when the AUC score is low.


$$\begin{array}{r} AUC\;=\;\frac{Specificity+Sensitivity}{2}\\ Sensitivity\;=\;Recall\;=\;\frac{TP}{TP+FN}\\ Specificity\;=\;\frac{TN}{FP+TN} \end{array}$$

Binary Crossentropy (3) is a cost function that gives the log distance between two probability distributions containing probabilities of an event occurring or not for any binary classification problem.


$$\begin{array}{r} Binary\;Crossentropy\;Loss\;=\;\frac{1}{\begin{array}{c} output\\ size \end{array}}\;\overset{\begin{array}{c} output\\ size \end{array}}{\underset{i=1}{\sum }}y_{i}\;.\;\log\hat{y_{i}}\\ +\left(1-y_{i}\right).\;\log\left(1-\hat{y_{i}}\right) \end{array}$$

### Model Comparison

**Figure 10:**
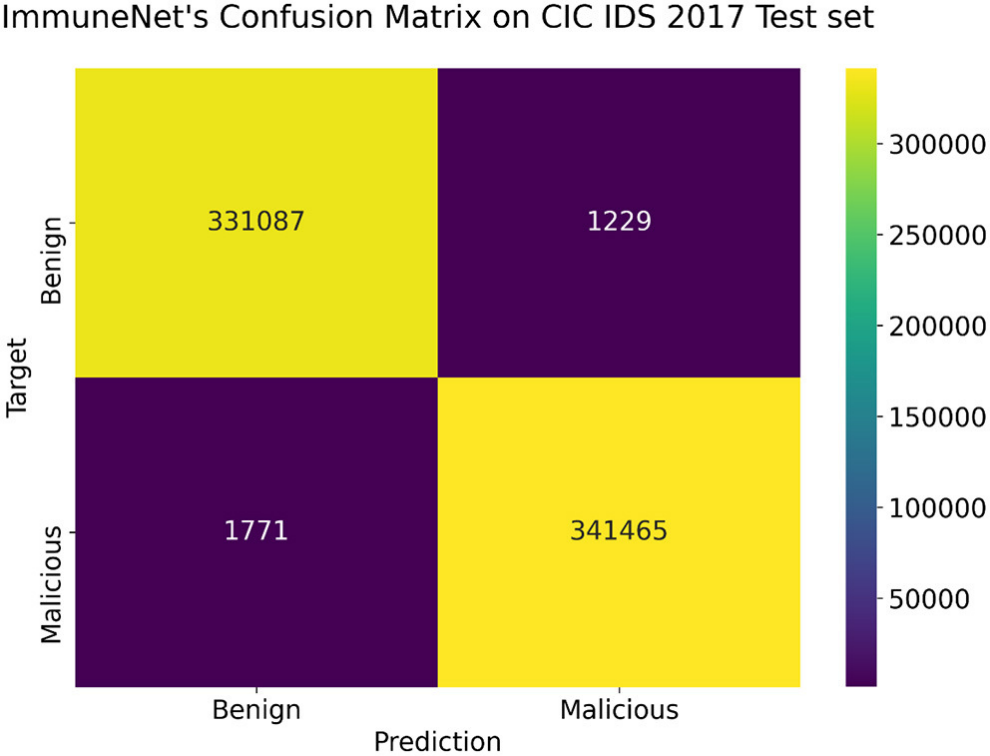
Confusion matrix on the CIC IDS 2017 test set for ImmuneNet.

**Figure 11:**
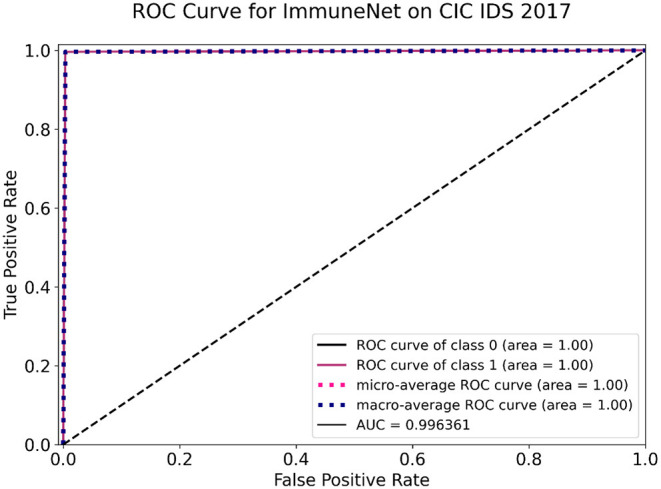
Receiver operating characteristic (ROC) curve of ImmuneNet on CIC IDS 2017 dataset.

The above models were tested on the test sets of CIC IDS 2017, CIC IDS 2018, and CIC IDS Bell DNS 2021 datasets, as mentioned in section Improving Class Balance. Table 8 contains the evaluation metrics of the algorithms' performance on the CIC IDS 2017 dataset. Figures 10, 11 display the confusion matrix and ROC curve of ImmuneNet on the CIC IDS 2017's test set.

**Table 8:** Comparison of models based on respective evaluation metrics for CIC IDS 2017.

**Model**	**Accuracy**	**Precision**	**Recall**	**F1**	**ROC-AUC Score**	**Training Loss**	**Validation Loss**	**Testing Loss**
ImmuneNet	99.63	99.64	99.63	99.63	99.636	0.0077	0.0104	0.0091
XGB	99.09	99.03	99.07	99.07	99.10	0.0150	0.0239	0.0216
Random forest	98.81	98.82	98.81	98.81	98.31	0.0388	0.0272	0.274
Decision trees	98.54	93.41	96.79	95.06	96.79	0.1132	0.0986	0.0961
Logistic regression	92.96	90.8	90.96	90.87	91.5	0.2341	0.2560	0.2663

From Table 8, we can observe that the Immune Net algorithm performs the best, with an accuracy of 99.63%. Compared to the other algorithms like XGB (12) and Random Forests, ImmuneNet has better accuracy and minimum loss. From the Confusion Matrix represented in Figure 10, we can also see that only 1,229 benign samples were flagged as malicious in total, implying a less false positive rate. On the other hand, XGB (12) and Random Forest perform better when compared to Decision Trees and Logistic Regression with an accuracy of 99.09 and 98.81 %.

The Receiver Operating Characteristic curve for ImmuneNet, as seen in Figure 11, has an AUC score of 99.6% for the CIC DIS 2017 test set. This makes it evident that the algorithm's true positive rate against the false positive rate is better and is almost a typically perfect binary classifier.

**Figure 12:**
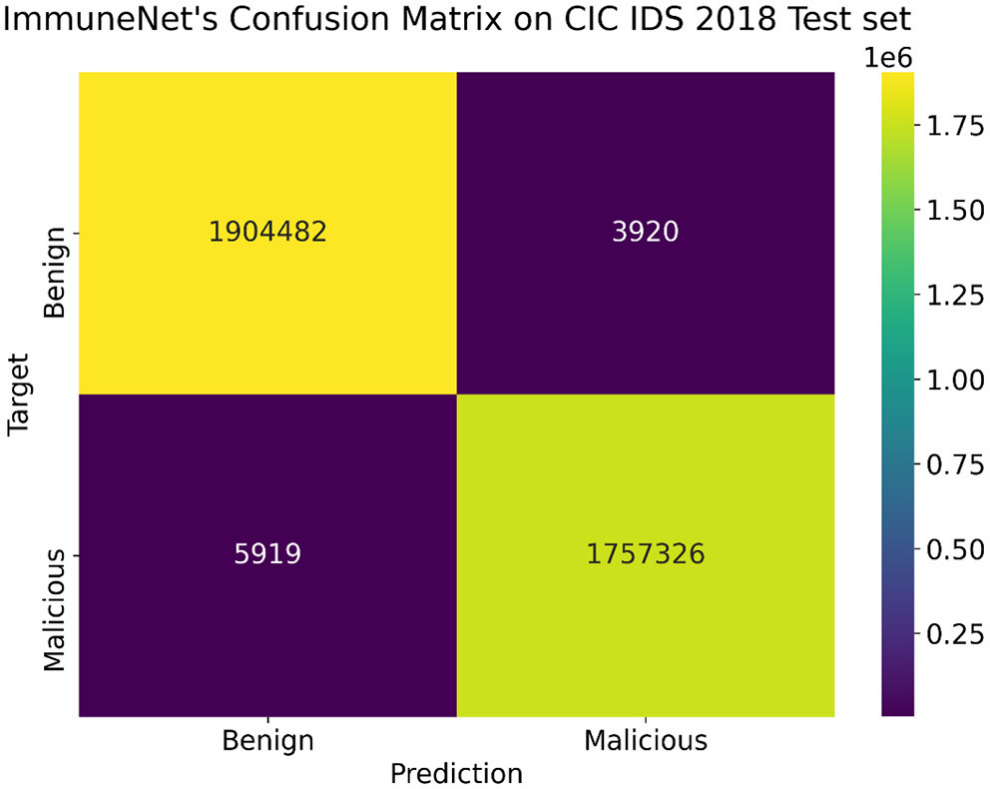
Confusion matrix on the CIC IDS 2018 test set for ImmuneNet.

**Figure 13:**
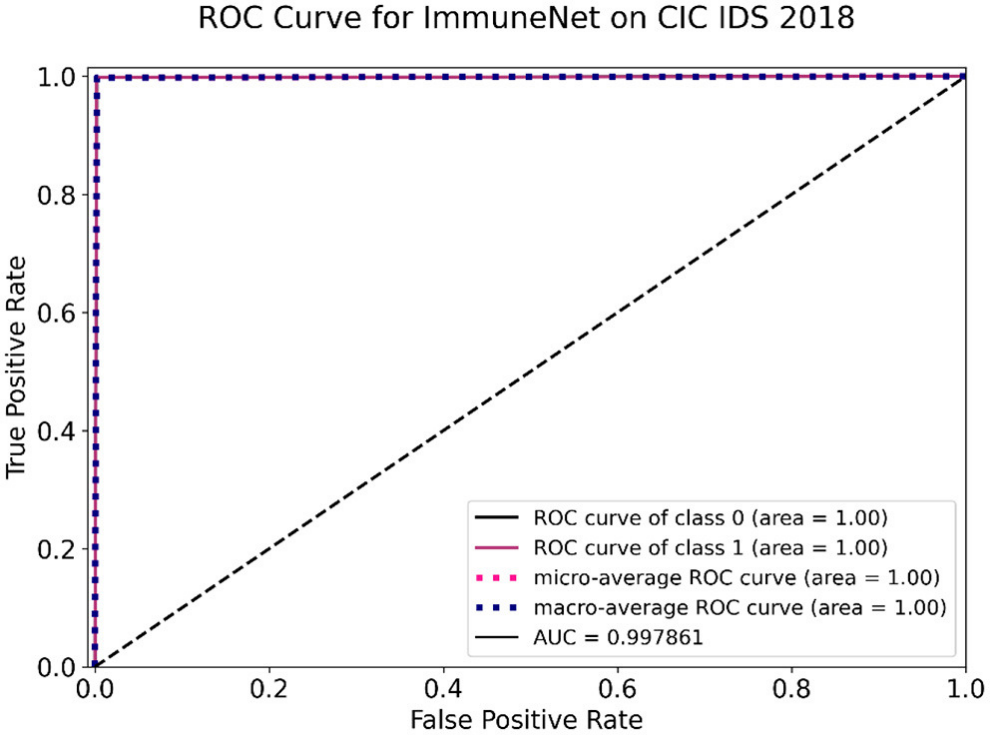
Receiver operating characteristic (ROC) curve of ImmuneNet on CIC IDS 2018 dataset.

Table 9 comprises the evaluation metrics of the five machine learning algorithms on the CIC IDS 2018 dataset. The confusion matrix and ROC curve of ImmuneNet are represented by Figures 12, 13 for the CIC IDS 2018's test set.

**Table 9:** Comparison of models based on respective evaluation metrics for CIC IDS 2018.

**Model**	**Accuracy**	**Precision**	**Recall**	**F1**	**ROC-AUC Score**	**Training loss**	**validation loss**	**Testing loss**
ImmuneNet	99.78	99.77	99.78	99.7	99.786	0.0036	0.0023	0.0025
XGB	99.00	99.03	99.00	99.01	98.98	0.0141	0.0366	0.0389
Random forest	98.81	98.82	98.81	98.81	98.31	0.0989	0.0782	0.0801
Decision trees	98.69	93.41	98.79	96.03	98.731	0.1054	0.1031	0.1053
Logistic regression	87.96	90.8	87.96	88.99	81.537	0.3520	0.2877	0.2798

ImmuneNet has an accuracy of 99.78, 99.77% precision score, 99.78% recall score, and a test loss of 0.0025, which is better when compared to the other algorithms. Logistic Regression had the lowest accuracy of 87.96% and had a high loss. Figure 12 depicts the Confusion Matrix, from which we can observe that the false positives and false negatives are very low, having only samples around 3,920 and 5,919, respectively.

From the ROC curve, as presented in Figure 13, we can observe that ImmuneNet's performance has a better True Positive rate against False Positive rate producing a ROC-AUC score of 99.78%. The obtained metrics are higher than other existing methods for CIC IDS 2018, such as the LighGBM and HBGB method proposed by Seth et al. (19).

**Figure 14:**
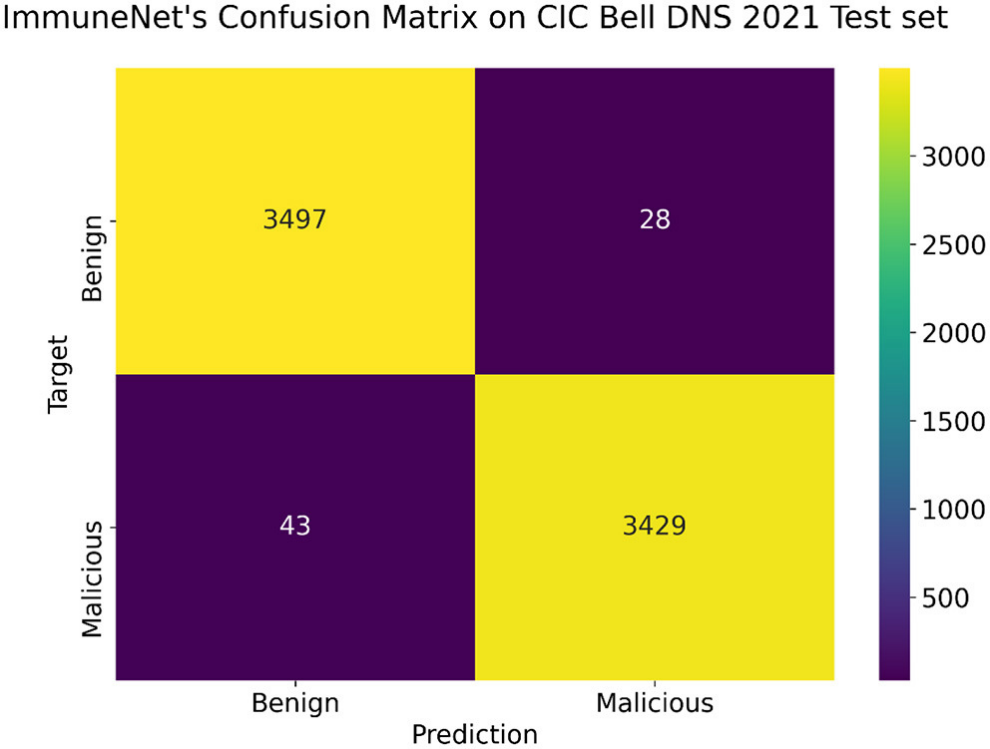
Confusion MATRIX on the CIC Bell DNS 2021 test set for ImmuneNet.

**Figure 15:**
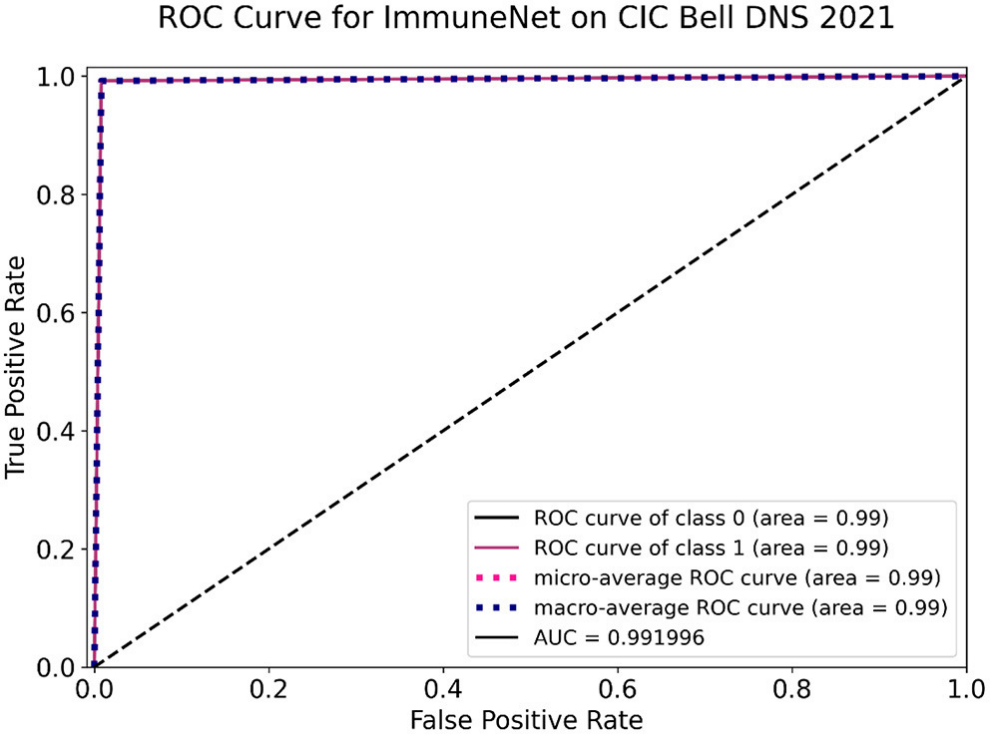
Receiver operating characteristic (ROC) curve of ImmuneNet on CIC Bell DNS 2021 dataset.

Several evaluation metrics in Table 10 depict the algorithms' performance on the CIC Bell DNS 2021 dataset. ImmuneNet's confusion matrix and ROC curve on the CIC Bell DNS 2021's test set are represented by Figures 14, 15.

**Table 10:** Comparison of models based on respective evaluation metrics for CIC Bell DNS 2021.

**Model**	**Accuracy**	**Precision**	**Recall**	**F1**	**ROC-AUC Score**	**Training Loss**	**Validation Loss**	**Testing Loss**
ImmuneNet	99.19	99.22	99.19	99.20	99.199	0.0124	0.0143	0.00141
XGB	99.10	98.89	99.10	98.99	99.01	0.0235	0.0228	0.0226
Random forest	98.21	95.60	98.17	96.86	96.25	0.0789	0.0631	0.0655
Decision trees	97.66	95.41	98.78	97.06	96.30	0.0808	0.0748	0.0752
Logistic regression	92.87	89.93	92.60	91.24	90.10	0.3356	0.3579	0.3467

From Table 10, it is observed that the ImmuneNet has the highest accuracy of about 99.2% and a test loss of 0.00141. These metrics are much better when compared with the algorithms like Decision Trees, Random Forest and Logistic Regression, which have comparatively lower accuracies and higher losses.

From Figure 14, we can see that ImmuneNet has an excellent true positive rate against false positive rate, having a ROC-AUC score of 99.19%. It is evident that ImmuneNet is performing better on the CIC Bell DNS 2021 dataset from the metrics mentioned above compared to Mahdavifar et al. (8). They achieved about 94.8 and 99.4% accuracy on the 60/40% balanced dataset and the 97/3% balanced dataset. The discussion on the performance of the algorithms mentioned above for all three datasets with their advantages and limitations is elaborated under the next section.

## Discussion

Out of all the experiments run on the CIC IDS 2017 (6), CIC IDS 2018 (7), and CIC Bell DNS 2021 (8) datasets with over five types of machine learning algorithms, ImmuneNet performed the best for all three datasets. This is because the algorithms' training, validation, and testing phases were data-centric and highly dependent on the class balancing and feature engineering techniques we followed for the three datasets. ImmuneNet achieved ~99.2% accuracy for the CIC Bell DNS 2021 dataset, 99.8% accuracy for CIC IDS 2018 dataset, and 99.63% accuracy for the CIC IDS 2017 dataset during the testing phase. These metrics are better than the existing approaches that have been suggested for IDS in healthcare, such as methods proposed by Subasi et al. (4), Šabić et al. (15), Hady et al. (16) since the model has been trained on an extensive real-time dataset containing latest cyberattacks post multiple feature engineering and sampling techniques. The algorithm has also performed well-after performing a different sampling and preprocessing approach for the CIC Bell DNS 2021 dataset, achieving an accuracy of about 99.2%, which is comparatively better than the method proposed by Samaneh Mahdavifar et al. (8).

The four reasons behind ImmuneNet's good performance are the data preprocessing, residual operation (38), mish activation (25), and layer normalization (39). By adding the tensors from the first layer and the tensors from the last layer, we preserve features learned by the first layer even if the features learned by the last layer were terrible. This also makes a difference during backward propagation. Mish activation (25) is unbounded above, allowing positive values and slight negative values to flow through the network without any saturation caused by capping during the forward and backward propagation. Layer normalization (39) normalizes the input tensor to zero mean and unit variance along the feature direction. The feature selection, synthetic sampling techniques, and data standardization during preprocessing have led to well-balanced and processed data from which the model was able to learn better.

**Figure 16:**
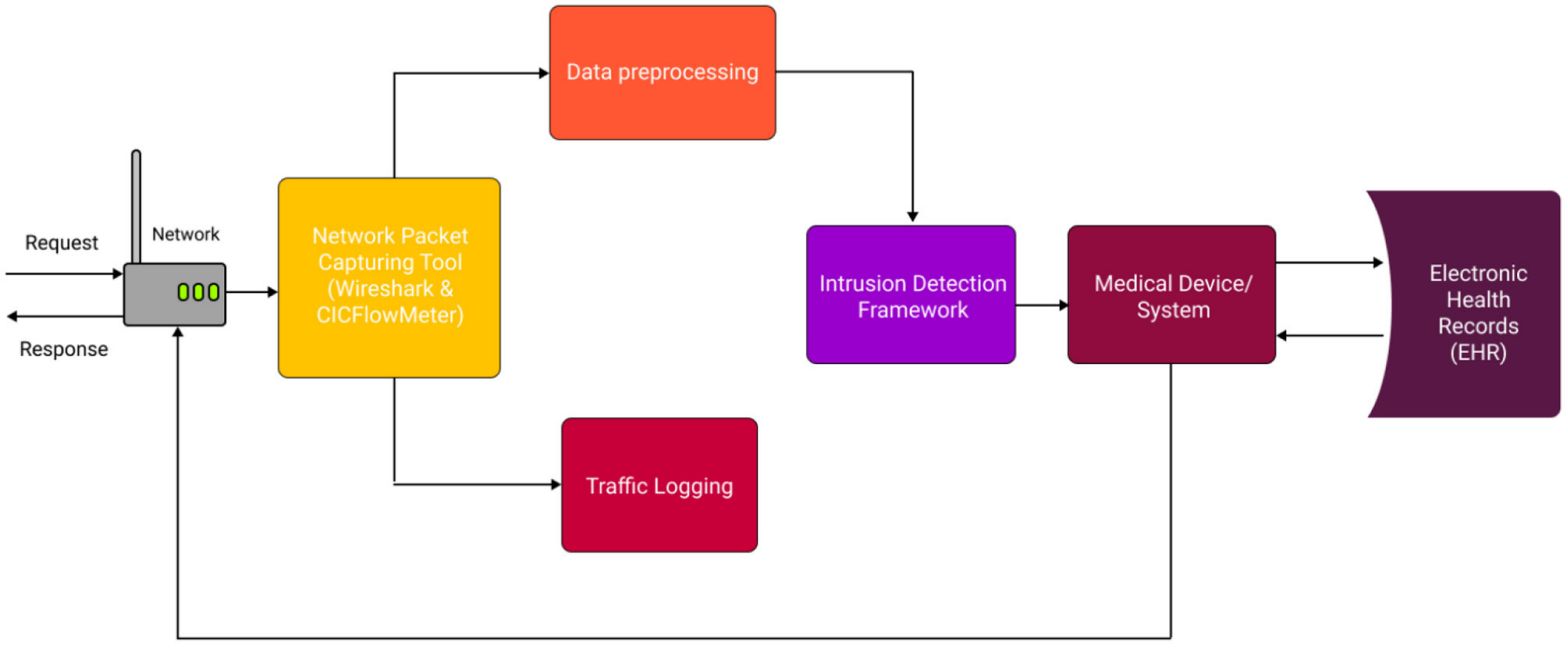
System architecture diagram for the proposed intrusion detection system.

Apart from ImmuneNet, other algorithms like XGB (12) and Random Forest also performed competitively, having the best subsequent metrics for all three datasets. The learning algorithms must be trained on balanced datasets to achieve better performance and unbiased detection of malicious requests. The CIC Bell DNS 2021 had a heavy class imbalance, and the number of samples obtained after the data preparation was low compared to CIC IDS 2017 (6) and 2018 (7) datasets. The CIC Bell DNS dataset can be improved by including more real-time samples for the phishing, spam, and malware domains to make it more balanced, and the algorithm can be fine-tuned to improve its performance. The ImmuneNet model is very lightweight, containing <1 million parameters. Table 11 displays the parameters and size of the model's weight in megabytes for all three architectures. This makes it easy to deploy it on any medical device or healthcare system without occupying much memory and would also be fast during inference and fine-tuning. However, the model has to be fine-tuned to support new types of attacks in the future to keep it abreast and safeguard healthcare systems with high accuracy. The neural network's weights can be frozen except the final classification layer, and the framework can be trained again with new data to fine-tune the model. The training process can be done again with new data to retrain the model and improve its performance. Figure 16 displays the system architecture of the proposed intrusion detection system showing the typical deployment setting and entire process flow.

**Table 11:** ImmuneNet's model parameters and size for CIC IDS 2017–2018, and CIC Bell DNS 2021 datasets.

**S. No**	**Dataset**	**Number of trainable parameters**	**Model weights size (in megabytes)**
1	CIC IDS 2017	828,209	3.265
2	CIC IDS 2018	828,209	3.265
3	CIC Bell DNS 2021	825,137	3.253

## Conclusion

The proposed neural network model, ImmuneNet, has given the highest accuracy of over 99.2% on the class balance improved CIC Bell DNS 2021 dataset, 99.8 and 99.63% accuracy on the oversampled CIC IDS 2018 and 2017 datasets. The model obtained a ROC-AUC score of 99.19%, which is considerably better than the score achieved by the other approaches. We followed a data-centric approach and used several feature selection and sampling techniques to improve class balance and the pre-processing of the datasets, leading to these results. This architecture is the latest in terms of Intrusion Detection as it has been trained with three latest real-time and extensive datasets. It is deployment-friendly for medical devices and healthcare systems because of its lightweight and fast characteristics. This would help protect the patient records from any kind of cyber attack, leading to consistency in patients' medical data. It is also highly critical that the models are not biased toward specific attacks since bias can allow certain attacks to bypass the IDS and manipulate the patient records by entering into the system. ImmuneNet has an excellent true positive rate against false positive rate, implying it is not biased toward false positives and negatives. However, the CIC DNS 2021 dataset needs to be improved with more samples from phishing, spam, and malware domains to promote natural class balance and better benchmarking of the algorithms. The proposed system must also be frequently fine-tuned with new cyber attack data to protect patient records from cyber attacks that emerge in the future. Fine-tuning the model on the latest cyber-attack data from time to time would improve the model's performance and keep it up-to-date, but it is a time-consuming and tedious process. To overcome these disadvantages, our future work would include a self-supervised learning-based neural network architecture that can make systems like intrusion detection adapt by themselves to new attacks without any manual retraining or fine-tuning and focusing on the recent datasets with better class balance and adversarial reweighting strategies to improve the fairness of the model.

## Data Availability Statement

The original contributions presented in the study are included in the article/supplementary material, further inquiries can be directed to the corresponding author.

## Author Contributions

C-YC did the conceptualization, supervised the research, and carried out the funding acquisition. MA and DS investigated the data, performed the methodology, and implemented the software code. KS and C-YC carried out the project administration, validated the results, reviewed, and edited the manuscript. MA, DS, PV, and HG wrote the manuscript. All authors contributed to the article and approved the submitted version.

## Funding

This research was partially funded by Intelligent Recognition Industry Service Research Center from the Featured Areas Research Center Program within the framework of the Higher Education Sprout Project by the Ministry of Education (MOE) in Taiwan and Ministry of Science and Technology in Taiwan (Grant No. MOST 109-2221-E-224-048-MY2).

## Conflict of Interest

The authors declare that the research was conducted in the absence of any commercial or financial relationships that could be construed as a potential conflict of interest.

## Publisher's Note

All claims expressed in this article are solely those of the authors and do not necessarily represent those of their affiliated organizations, or those of the publisher, the editors and the reviewers. Any product that may be evaluated in this article, or claim that may be made by its manufacturer, is not guaranteed or endorsed by the publisher.
